# Temperament and personality: preliminary evidence of possible relationships with multifactorial stress reactivity in healthy adolescents

**DOI:** 10.3389/fpsyg.2025.1613000

**Published:** 2025-07-22

**Authors:** Angelika Ecker, Irina Jarvers, Ricarda Jacob, Stephanie Kandsperger, Romuald Brunner, Daniel Schleicher

**Affiliations:** Department of Child and Adolescent Psychiatry and Psychotherapy, University of Regensburg, Regensburg, Germany

**Keywords:** adolescents, stress, personality, temperament, cortisol, alpha-amylase, subjective stress

## Abstract

**Objective:**

It is hypothesized that personality and temperament influence the stress response. However, no study has thoroughly investigated the impact of these factors during adolescence, a critical stage of development and consolidation. In this study, we aimed to explore this relationship, both for personality and temperament aspects, in a sample of adolescents. Therefore, an experimental stress induction, combined with multifactorial stress assessment, incorporating both biological and subjective measures, was conducted.

**Method:**

An acute psychosocial stress reaction was induced in 73 healthy adolescents (11–17 years of age, 63.0% female). Features of the stress response were recorded, including salivary cortisol, salivary alpha-amylase, heart rate, heart rate variability, and subjective stress. We investigated relationships between these factors and control variables (e.g., stress vulnerability and traumatic life experiences), specific trait facets, personality profiles according to the Big Five, and temperament dimensions according to Cloninger.

**Results:**

In bivariate correlations, salivary cortisol response was negatively associated with Extraversion. Regarding bivariate correlations with temperament, Harm Avoidance was particularly associated with cortisol response and with the subjective stress response. Only stress vulnerability was significantly related to the subjective stress response.

**Conclusion:**

In conclusion, the associations between personality/temperament profiles with the stress response are already evident during adolescence, highlighting the developmental aspect and the early emergence of these relationships. These findings suggest that personality and temperament profiles relate to individual differences in adolescent stress sensitivity. Identifying profiles linked to heightened or prolonged stress responses—such as high harm avoidance—may inform early interventions to support at-risk youth.

## 1 Introduction

Stress is widely recognized as a major risk factor for both physical and mental health (Yaribeygi et al., 2017; Špiljak et al., 2022), making it essential to better understand its underlying mechanisms. Depending on the perspective, stress can be conceptualized in different dimensions. Stress has different dimensions, depending on the perspective. From a biological perspective, stress arises as a result of the body's physiological response to challenges. Allostatic load refers to the cumulative, long-term effects of this response in adapting to internal and external demands (McEwen, 1998). Allostasis, in contrast, describes the process by which the body maintains stability through change and is primarily regulated by two systems: the stress hormone cortisol is released via activation of the hypothalamic-pituitary-adrenal (HPA) axis, and noradrenaline and adrenaline are rapidly released via activation of the sympathetic-adrenal-medullary (SAM) system, which also activates the cardiovascular system (O'Connor et al., 2021). These systems enable an organism to automatically react to stress.

According to the transactional model of stress and coping, psychological stress arises through the cognitive processes of appraising a situation and the available resources (Lazarus and Folkman, 1984). The transdisciplinary model of stress combines different dimensions—positing that experiences, environments, and individual dispositions influence the psychological perception of and physiological reactions to a stressor (Epel et al., 2018). Individual dispositions include personality, i.e., “the enduring configuration of characteristics and behavior that comprises an individual's unique adjustment to life, including major traits, interests, drives, values, self-concept, abilities, and emotional patterns” (American Psychological Association, 2024a). Different stress reactions can be attributed to an individual's personality (Epel et al., 2018), which here refers to the widely accepted five-factor model (Big Five) by Costa and McCrae (1992)—including Neuroticism, Extraversion, Agreeableness, Conscientiousness, and Openness.

In a recent meta-analysis, Luo et al. (2023) analyzed the relationship between personality and stress reactions, elucidating a positive correlation between psychological stress reactivity and Neuroticism, along with significant but weaker negative correlations between other personality traits and subjective stress reactivity. Various measures were used as indicators of psychological stress reactions—including single-item questions regarding experienced stress, time pressure, hassles, and other aspects, such as job demands or role overload. The measured physiological parameters included cortisol, heart rate, heart rate variability, blood pressure, and skin conductance, which generally showed weak to null associations with the Big Five personality traits. However, a weak but notable negative correlation was observed specifically between Extraversion and some physiological parameters (e.g., cortisol, cardiovascular indicators). Moreover, the results of this meta-analysis suggested that personality traits are more strongly associated with the cardiovascular stress response (heart rate, diastolic blood pressure, and systolic blood pressure) compared to other physiological stress parameters, such as cortisol, heart rate variability, and skin conductance (Luo et al., 2023). However, few studies have analyzed multiple physiological stress parameters and investigated potential relationships to personality traits. Therefore, while it appears that the stress response is somewhat related to individual personality profiles, it is unclear to what extent the stress response is linked personality profiles. This highlights the importance of examining this topic using acute stress induction and multimodal stress assessment.

Apart from the Big Five personality model, other models have been investigated for potential association with stress, including temperament, which is defined as “the basic foundation of personality, usually assumed to be biologically determined and present early in life, including such characteristics as energy level, emotional responsiveness, demeanor, mood, response tempo, behavioral inhibition, and willingness to explore” (American Psychological Association, 2024b). Different aspects of temperament—Novelty Seeking, Harm Avoidance, Reward Dependence, and Persistence—are represented in Cloninger's temperament dimension (Cloninger et al., 1993) and are relevant in an individual's stress reaction, particularly Novelty Seeking and Harm Avoidance, which is linked to emotional reactions to tasks (Puttonen et al., 2005). These two temperament dimensions are also reportedly inversely correlated with the stress response, measured via cortisol (Tyrka et al., 2007, 2008). Additionally, temperament dimensions have been linked to subjective stress appraisals, that is, how individuals perceive and evaluate challenging situations as stressful, which in turn can influence stress vulnerability (Ravaja et al., 2006). This indicates a correlation between stress reactivity and temperament, which has been explored in fewer studies compared to the Big Five personality model.

Interesting connections have also been reported between the stress reaction and specific trait facets. A trait is defined as “an enduring personality characteristic that describes or determines an individual's behavior across a range of situations” (American Psychological Association, 2024c). One such construct is alexithymia, which is characterized by a deficit in emotion processing, and exhibits a normal distribution in the population (Parker et al., 2008). Alexithymia severity has been positively associated with HPA axis stress reactivity, suggesting that individuals with higher alexithymia levels may show stronger physiological responses to stress (Hua et al., 2014). Another construct is empathy, which may be linked to physiological and psychological stress responses, with lower empathy reportedly related to reduced stress responses (Fairchild et al., 2019; Laviola et al., 2017; Tollenaar and Overgaauw, 2020). Similarly, the trait impulsivity is positively associated with the cardiovascular response (Allen et al., 2009; Bibbey et al., 2016). A related construct is the aspect of aggression, and higher aggression is reportedly linked to lower blood pressure in response to stress (Flaa et al., 2007). In contrast to these externalizing factors, the internalizing domain includes anxiety and depression traits, which both seem to be correlated with a decreased stress reaction (Jezova et al., 2004; Rooij et al., 2010). Overall, specific trait facets appear to be associated with the individual stress response.

On the whole, the available evidence indicates that specific trait facets, personality domains (like Neuroticism and Extraversion), and temperament dimensions (such as Novelty Seeking and Harm Avoidance) are of particular importance for understanding the stress response. However, existing studies have focused exclusively on adults. Stress reactivity at a young age is relevant for psychiatric conditions, such as depression, or cardiovascular diseases in the long-term (Hankin et al., 2015; O'Connor et al., 2021). Adolescence is a vulnerable period of central nervous system development, which influences the reward system, avoidance and withdrawal behavior, and self-regulation (Eldreth et al., 2013). Moreover, both personality (McCrae et al., 2002) and temperament (Zohar et al., 2019) undergo changes during adolescence. It is of great interest to examine the relationship between personality/temperament and stress reactivity during a phase of change and stabilization. While it is still unclear how valid the associations between certain personality traits and stress reactivity are, given that temperament and personality may change during adolescence, our focus is on understanding the multimodal stress response during this dynamic period. In the present study, we aimed to exploratively investigate relationships between an acute multifactorial stress reaction and personality and temperament profiles, as well as specific trait facets (i.e., alexithymia, empathy, impulsivity, aggression, and trait anxiety and depression), of healthy adolescents, and to examine whether these profiles can explain a significant proportion of variance of the stress reactivity.

## 2 Methods

### 2.1 Design

All participants underwent stress induction via two versions of the Trier Social Stress Test (TSST; original protocol (*n* = 37) or virtual setting (*n* = 36). Then their stress responses were analyzed in relation to their personality/temperament aspects and specific trait facets. This study was conducted as part of a larger project investigating the core topics of stress induction and subsequent relaxation, with two strategies used for each. Following the TSST, participants underwent a short relaxation period (5 min), during which they were randomly assigned to either a guided breathing exercise or an unguided rest condition. However, previous analyses revealed no significant effects of the relaxation condition on acute stress reactivity (Schleicher et al., 2024), which is the focus of the present study. Therefore, under the current research question, all subgroups were considered together. Detailed information regarding the larger project is available in the published study protocol (Schleicher et al., 2022). Therefore, the methods section in the current manuscript was deliberately kept concise and focused on aspects most relevant to the specific research questions addressed in this secondary analysis.

### 2.2 Participants

Inclusion criteria were age between 11 and 17 years, and sufficient understanding of the German language. Exclusion criteria to rule out confounding influences were current or past psychiatric, psychotherapeutic, or neurological treatments; use of medication containing glucocorticoids; and the presence of mental, neurological, endocrinological, or immunological (pre)disorders. Additional exclusion criteria were pregnancy, breastfeeding, early pubertal development, intellectual disability, or attendance at a special school. Recruitment was primarily conducted via email distribution lists, posters, and flyers targeting employees of various local hospitals and their children.

A total of 84 adolescents were recruited. Of these, seven were no longer motivated to participate before the study began, three were excluded due to suspected mental (pre-)disorders, and one dropped out during the study due to lack of adherence. Thus, the final sample included 73 adolescents (63.0% female), with an average age of around 14 years (*M* = 13.85 years; *SD* = 1.91 years). Participants had a high level of education, measured according to type of school (78.1%). Table 1 provides an overview of demographic characteristics. This study was approved by the Ethics Committee of the University of Regensburg (No.: 20–1800–101). All participants and their legal guardians gave written informed consent. Participants received a gift voucher worth €40–€50 for their participation.

**Table 1:** Sociodemographic characteristics and puberty status.

	***M (SD)***	**Range**
Age in years	13.85 (1.91)	11–17
	***N***	**%**
**Sex**		
Female	46	63.0
Male	27	37.0
**School type**		
Mittelschule	2	2.7
Realschule	13	17.8
Gymnasium	54	74.0
FOS/BOS	3	4.1
Berufsschule	1	1.4
**Puberty status**		
Prepubertal	7	8.5
Early pubertal	6	7.3
Midpubertal	18	22.0
Late pubertal	34	40.2
Postpubertal	9	11.0
Hormonal contraceptive intake (female)	1	1.4

All participants were asked about sex and gender, which were congruent in all cases. School types in Germany: Mittelschule, 9 years of elementary school; Realschule, intermediate level of secondary school, regular duration of 6 years; Gymnasium, highest level of secondary school, regular duration of 8–9 years; FOS (Fachoberschule)/BOS (Berufsoberschule), tertiary school to achieve advanced technical college certificate, duration of 2–3 years in addition to duration of Realschule; Berufsschule: vocational school, regular duration of 2–3 years, after secondary school.

### 2.3 Measures

#### 2.3.1 Personality–Big Five Inventory-10

The Big Five Inventory-10 (BFI-10) (Rammstedt and John, 2007) was used to assess personality across the established Big Five dimensions: Neuroticism (N), Extraversion (E), Openness (O), Agreeableness (A) and Conscientiousness (C). Each dimension is assessed based on one positive and one negative item, using a five-point Likert scale, ranging from 1 (strongly disagree) to 5 (strongly agree). The German version of the BFI-10 has demonstrated sufficient retest reliability (*r* = 0.58–0.84), factorial validity, and construct validity (*r* = 0.69) (Rammstedt et al., 2017). We assessed the internal consistency of each BFI-10 two-item subscale in our sample. Inter-item Pearson correlations were *r* = 0.40 for Extraversion, *r* = 0.31 for Agreeableness, *r* = 0.39 for Conscientiousness, *r* = 0.45 for Neuroticism, and *r* = 0.35 for Openness (all *p* < 0.01). Corresponding Cronbach's α coefficients were α = 0.57 (Extraversion), α = 0.48 (Agreeableness), α = 0.55 (Conscientiousness), α = 0.62 (Neuroticism), and α = 0.51 (Openness). These values—typical for an ultra-brief scale—are in line with those reported by Rammstedt and John (2007), reflecting the trade-off between brevity and internal consistency. Notably, the BFI-10 is designed for German-speaking individuals over 18 years of age, such that its norms are inapplicable to the adolescent sample. However, the short items, their relatively small number, and the simple wording make it feasible to use the BFI-10 with adolescents, as supported by other research (Muhametjanova et al., 2023).

#### 2.3.2 Junior Temperament and Character Inventory (JTCI 12-18 R)

The Junior Temperament and Character Inventory is a self-report questionnaire designed to measure the personality aspects of “temperament” and “character” in adolescents (Goth and Schmeck, 2009). The questionnaire comprises 103 items answered using a five-point Likert scale, ranging from 0 (no) to 4 (yes). Since character is more related to a person's values, we considered only temperament in the present study. Temperament is assessed using the four scales—Novelty seeking (NS), Harm Avoidance (HA), Reward Dependence (RD), and Persistence (P)—which are divided into four subscales each. The inventory is intended for use by individuals aged 12–18 years, although it is considered acceptable for use by participants outside this range. Its reliability has been confirmed based on internal consistency (Cronbach's α = 0.79 and α = 0.85), as was the factor structure and criterion validity according to related personality measures (Goth and Schmeck, 2009). Comparable internal consistency coefficients were found in our sample, with Cronbach's α ranging from 0.77 to 0.87, indicating similarly high reliability.

#### 2.3.3 Specific trait facets

Several constructs are considered specific trait facets, and we investigated a few of these for their relationship to stress reactivity in adolescents. The Alexithymia Questionnaire for Children (AQC) in English (Rieffe et al., 2006) was developed from the Toronto Alexithymia Scale (TAS-20; Bagby et al., 1994), and has been translated into German (AQC-G) (Jarvers et al., 2022). The AQC-G comprises 20 items, some of which are inverted, and measures the overall scale “alexithymia”, as well as three subscales: “difficulties in recognizing emotions”, “difficulties in describing emotions”, and “externally oriented thinking”. Items are answered using a three-point Likert scale, ranging from 0 (not true) to 2 (often true). The AQC-G is designed for children aged 9–15 years, covering the majority of the age range in this study. The questions can also be answered by older participants, as the adaptations for children focus on comprehensible language rather than age-specific examples. The English version has shown good psychometric properties, with the exception of the “externally orientated thinking” subscale, which exhibits poor reliability and factor structure (Rieffe et al., 2006). The German version is currently undergoing validation, with no results yet available. In our present sample, the internal consistency was very similar, with Cronbach's α = 0.77 for the overall scale, and appropriate ranges for the subscales, with the lowest values for externally orientated thinking (α = 0.51–0.82).

Empathy as a personality trait can be assessed using the Cognitive, Affective, and Somatic Empathy Scales (CASES) (Raine and Chen, 2018). Here we used its German version (CASES-G) (Schleicher et al., 2021). This self-report questionnaire distinguishes between cognitive and affective empathy, and also encompasses somatic empathy and accounts for empathy for negative and positive emotions (Raine and Chen, 2018), resulting in a total of six factors. The questionnaire comprises 30 items, answered using a three-point Likert scale, with options of 0 (rarely), 1 (sometimes), and 2 (often). The total score can range from 0–60, with higher values indicating higher empathy. The original version was constructed for 11-year-old children. However, the same wording has been validated for use in adulthood (Raine et al., 2022), making it particularly suitable for the present sample. The original version shows excellent internal consistency (α = 0.91), and its validity has been confirmed. The German translation is currently being validated. It showed good internal consistency in the present sample (α = 0.89).

The State-Trait Anxiety-Depression Inventory (Laux et al., 2013) is a self-assessment scale that can be used to differentiate between anxiety and depression at both the state and trait levels. For this study, only the trait scale was used for analysis. The trait is assessed using 20 questions answered using a four-point Likert scale, ranging from 1 (almost never) to 4 (almost always), with partially inverted items. The total score ranges from 20–80 points. While the STADI is recommended for individuals of at least 16 years of age, the German version has been successfully used in adolescents as young as 13 years (Matulis et al., 2015), indicating its potential application among a younger sample. Reliability of the anxiety and depression scales, both state and trait, falls within an appropriate range (α = 0.87–0.90). Validity testing confirmed the convergent and discriminant correlations, as well as the factorial validity of the questionnaire (Laux et al., 2013).

The Barratt Impulsiveness Scale–short version (BIS-15) (Spinella, 2007) was used in its German version (Meule et al., 2011) to measure impulsivity as a personality trait. This questionnaire comprises 15 items rated on a four-point Likert scale, ranging from 1 (rarely/never) to 4 (almost always/always). The total score ranges from 15–60, with higher values indicating higher impulsivity. The applicability to children and adolescents has been demonstrated in various studies, covering a wide age range (Bhat et al., 2018; Fossati et al., 2002; Kattein et al., 2021). The questionnaire was understandable even by eight-year-olds (Cosi et al., 2008), suggesting that the total score can be interpreted without restriction. The German version of the BIS-15 shows good internal consistency (α = 0.81) and its convergent validity has been confirmed (Meule et al., 2011).

Aggression was assessed using the self-report Aggression Questionnaire (AQ) (Buss and Perry, 1992) in its German translation (Werner and Collani, 2004). The AQ comprises 29 items, each rated on a four-point Likert scale, ranging from 1 (strongly disagree) to 4 (strongly agree), with scale values calculated through aggregation. The four scales—physical aggressiveness, verbal aggressiveness, anger, and hostility—can also be summarized to form a total score, which is used in the present study. The AQ was originally designed for use in adults, but has also been used with children and adolescents, with even nine-year-olds having no comprehension problems (Santisteban et al., 2007). The internal consistency of the subscales has been deemed appropriate (α = 0.62–0.82), and validity has been primarily confirmed by differential validity (Werner and Collani, 2004).

#### 2.3.4 Exclusion and control variables

To ensure the study sample was free of psychiatric disturbances, which could confound profiles or stress reactivity, the exclusion criterion of mental disorders was explicitly checked. This was accomplished by using the German version of the Mini-International Neuropsychiatric Interview for Children and Adolescents (M.I.N.I. KID 6.0) (Sheehan et al., 2010), and by screening for current or past neuropsychiatric disorders according to DSM-IV and ICD-10.

Pubertal status appears to influence physical aspects of the stress response, including cortisol, alpha-amylase, and heart rate (Cicone et al., 2019; Netherton et al., 2004; van den Bos et al., 2014), and thus must be monitored. To this end, we utilized the German version of the pubertal development scale (PDS) (Petersen et al., 1988; Watzlawik, 2009). This self-report questionnaire contains sex-specific inquiries related to pubic hair, beard/breast growth, and voice change/menarche. Responses are rated on a four-point Likert scale, ranging from 1 (has not yet started) to 4 (corresponds to an adult woman/man). The menarche question is a simple yes/no query. These evaluations enable determination of an ordinal puberty status, and the German version yields reliable and valid results (Watzlawik, 2009).

Underlying traumatization is another factor that could influence cortisol (Lai et al., 2020) and alpha-amylase (Schumacher et al., 2022) in the stress response. We accounted for this by using the German version of the Childhood Trauma Questionnaire–Short Version (CTQ-SF) (Bader et al., 2009; Bernstein et al., 2003). This retrospective questionnaire contains 28 items, each assessed using a five-point Likert scale, ranging from 1 (not at all) to 5 (very often). The questions evaluate five potential areas of trauma (emotional, physical, and sexual abuse; and emotional and physical neglect) with good reliability and validity (Bernstein et al., 2003) and can be summarized to generate a general index of adverse early life experiences.

To control for individual subjective vulnerability to stress, we used the first part of the Questionnaire for the Measurement of Stress and Coping in Children and Adolescents 3–8 R (SSKJ 3–8 R) (Lohaus et al., 2006). The scale “stress vulnerability” comprises seven items that are answered using a four-point Likert scale, ranging from 1 (no stress at all) to 4 (a lot of stress), represented as emojis. Answers are combined to generate a score, and this subscale demonstrates good reliability and validity (Lohaus et al., 2006).

### 2.4 Stress induction and parameters

Stress was induced using the Trier Social Stress Test (TSST) (Kirschbaum et al., 1993) tailored to the age of the sample (Buske-Kirschbaum et al., 1997). Here, the participants stand in front of a panel comprising a man and a woman, and are instructed to present themselves in a positive light, while the panel members give only neutral feedback, which is usually experienced as negative. The participants are given instructions on applying for the position of student representative, and on how to convince the panel of one's qualifications within three 5-min sections: a preparation phase for the speech, a speech phase, and a calculation phase during which they must calculate backward aloud, and without errors. In the context of a different research question, one part of the sample underwent the TSST in a virtual environment, while the other group faced a real panel. Group allocation was randomized, and both groups exhibited a significant stress response on all stress parameters (Ecker et al., 2024). Therefore, this condition had no influence on the presently investigated topic.

Various parameters were recorded to assess the multifactorial stress reaction. As the HPA axis parameter, salivary cortisol was measured using salivettes (Hellhammer et al., 2009). As a stress parameter of the autonomic nervous system, alpha-amylase was also measured using salivettes (Nater and Rohleder, 2009). Saliva samples were collected immediately before the TSST (0 min), immediately after the TSST (20 min), and then at 25, 30, 40, 60, and 80 min. Another autonomic nervous system parameter was the heart rate (HR) (Nater and Rohleder, 2009), which was recorded using an electrocardiogram and activity sensor “EcgMove 4” (movisens, Munich, Germany), and preprocessed using the software “DataAnalyzer” (version 1.13.5; movisens, Munich, Germany). From these recordings, we also determined the heart rate variability (HRV), another parameter of the autonomic nervous system. We calculated the root mean square of successive differences (RMSSD), which is a time-specific HRV marker that can be used to record short-term changes (Malik et al., 1996). HR and HRV were averaged in 5-min segments via the TSST periods.

In addition to the physiological stress reaction, the psychological response to stress was also recorded in the form of a subjective query. Participants were asked to respond to the prompt “At this moment, I feel stressed, tensed, or burdened” using a visual analogue scale (VAS) with ten levels, ranging from 1 (not at all) to 10 (very much). VAS responses were collected at all saliva sampling time-points, as well as after TSST instruction and before the preparation phase (5 min).

### 2.5 Procedure

**Figure 1:**
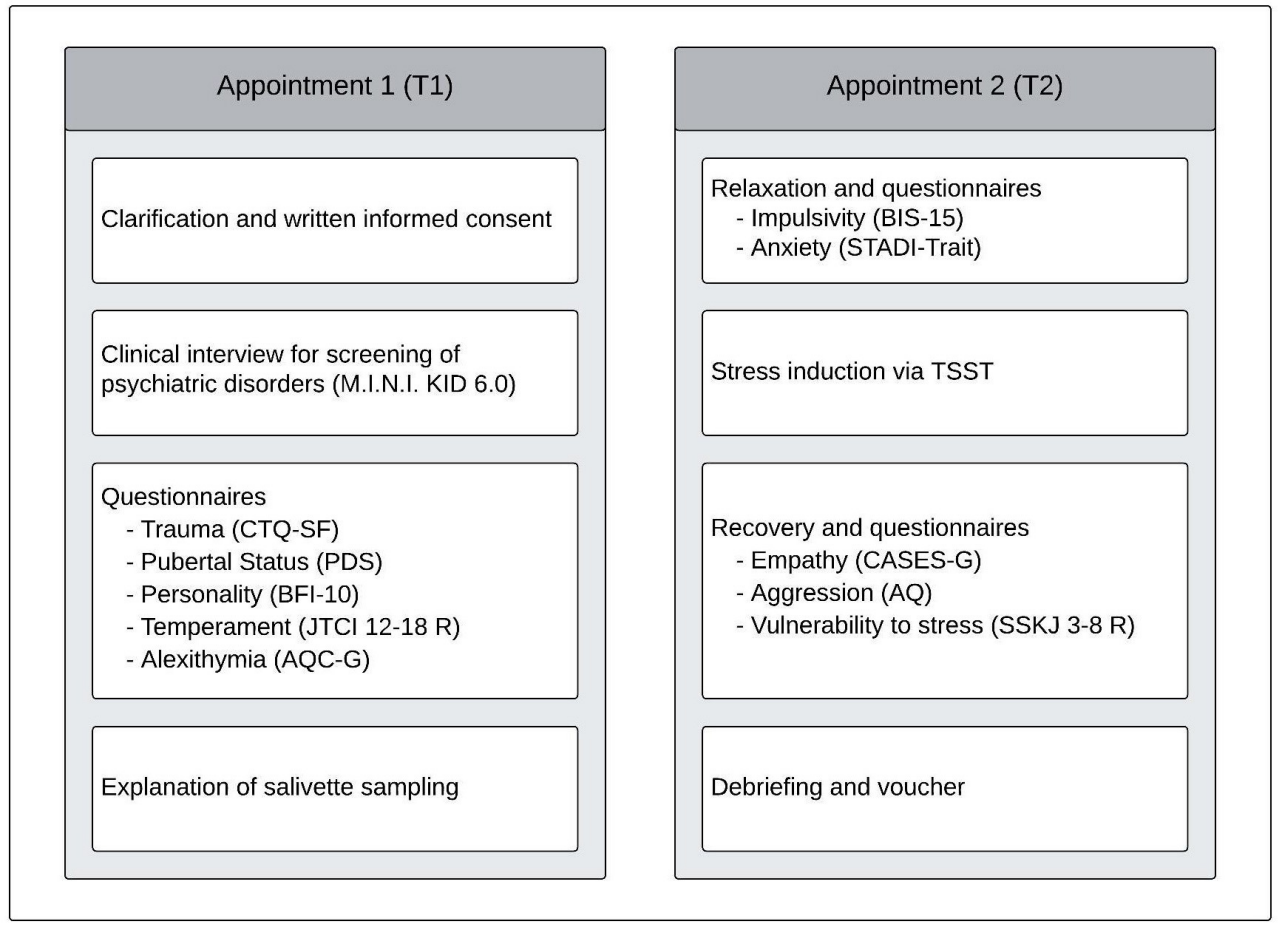
Overview of procedures at the first and second appointment. M.I.N.I. KID 6.0, mini international neuropsychiatric interview, 6th version; CTQ-SF, childhood trauma questionnaire–short form; PDS, pubertal development scale; BFI-10, big five inventory; JTCI 12–18 R, junior temperament and character inventory; AQC-G, alexithymia questionnaire for children, German version; BIS-15, barratt impulsiveness scale–short version; STADI, state-trait anxiety-depression inventory; TSST, trier social stress test; CASES-G, cognitive, affective, and somatic empathy scales, German version; AQ, aggression questionnaire, German version; SSKJ 3-8 R, questionnaire for the measurement of stress and coping in children and adolescents 3–8 R.

**Figure 2:**
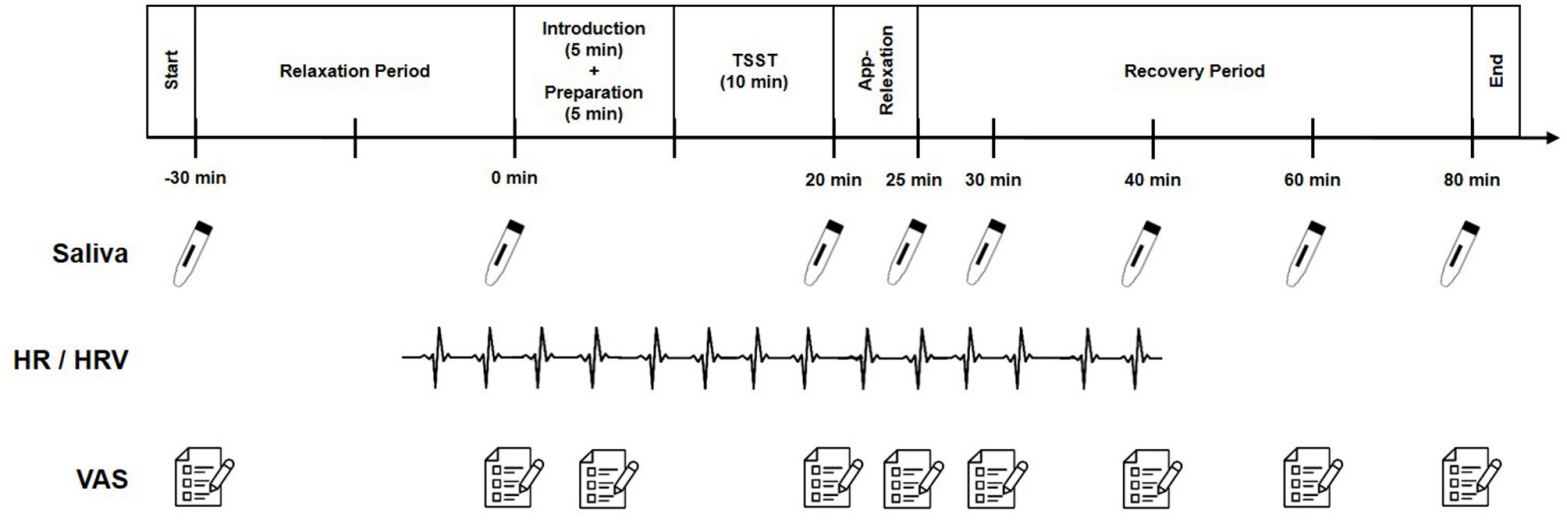
Overview of measurement time points for stress parameters across testing phases. TSST, trier social stress test; Saliva, saliva samples for cortisol and alpha-amylase; HR/HRV, heart rate/heart rate variability measures; VAS, visual analogue scale.

This study was carried out at the Clinic of Child and Adolescent Psychiatry, Psychosomatics and Psychotherapy, University of Regensburg, Germany. Participants attended two sessions. During the initial session (T1), participants and their legal guardian provided written informed consent, after which participants were tested without their guardian present. T1 lasted approximately 2.5 h, and involved a diagnostic interview, personality and temperament assessments, and various questionnaires. The second session (T2) involved stress induction through the TSST and the completion of additional questionnaires, and lasted 2 h due to the time-regulated protocol. Figure 1 presents the detailed procedures for T1 and T2, including a sequence-based presentation of the questionnaires. In addition to the described questionnaires, other aspects were surveyed, which are not the focus of the current research question. Figure 2 illustrates the measurement time points for the stress parameters during T2. The study protocol provides a comprehensive description of all methods and used questionnaires (Schleicher et al., 2022). Female participants who were menstruating were scheduled for T2 during their luteal phase, to control for the effect of menstrual cycle on the stress response (Montero-López et al., 2018). To ensure accuracy, 30.0% of the data were independently double-coded by another coder. Some stress parameters were analyzed in only a portion of the sample, due to technical issues in recording or extracting HR (*n* = 5) and HRV (*n* = 11), and laboratory problems with alpha-amylase extraction (*n* = 2). There were no missing values for cortisol or VAS. Some participants were missing questionnaire data (BFI-10: *n* = 1; AQC-G: *n* = 1; CTQ-SF: *n* = 2; and AQ: *n* = 2) because some questionnaires were either not filled out at all, or were insufficiently complete to be included in analysis.

### 2.6 Data preprocessing

The pre-processing of the stress parameters involved the following steps. Saliva samples were frozen and stored at −20° Celsius until analysis. Salivary concentrations of cortisol were measured using a commercially available highly sensitive chemiluminescence immunoassay (catalogue number R62111; Tecan - IBL International, Hamburg, Germany). The intra- and interassay coefficients of variance for cortisol were below 9%. Salivary concentrations of alpha-amylase were measured using an enzyme kinetic method. The intra- and interassay coefficients for alpha-amylase were below 5 and 9%, respectively.

To determine the area under the curve with respect to increase (AUCi) and ground (AUCg) (Pruessner et al., 2003), we used measurements from 0–80 min for cortisol (CORT AUCi / AUCg), and from 0–25 min for alpha-amylase (AMYL AUCi / AUCg. In addition to the parameters regarding the time-course (AUCi and AUCg), we calculated delta values for all stress parameters (maximum/minimum value – baseline), since they represent the maximum increase/decrease. We used the maximum increase in the time range of 20–40 min after TSST for cortisol (CORT Delta_increase_), 0–20 min for alpha-amylase (AMYL Delta_increase_), the maximum increase in the phases of TSST for heart rate (HR Delta_increase_), and the maximum decrease in the phases of TSST for HRV (HRV Delta_decrease_). For the subjective stress response (VAS), we calculated the delta for the anticipatory stress (5 min; VAS_Prep_ Delta_increase_) and actual stress induction (20 min; VAS_Stress_ Delta_increase_), using the baseline as the reference. This approach was chosen to capture and examine stress reactivity in response to stress induction; therefore, stress recovery was not within the scope of our processing and analyses.

### 2.7 Statistical analyses

We used Kendall's τ as correlation coefficient to account for non-normality and to best address potential outliers (Hollander, 2013). The correlation coefficient was interpreted as the effect size, with 0.1–0.3 indicating a small effect, 0.3–0.5 an intermediate effect, and ≥0.5 a strong effect (Cohen, 1988). Significant uncorrected correlations with at least a small effect size were further investigated using linear regressions, in which the respective stress parameter was the dependent variable. The independent variables comprised the relevant personality or temperament dimensions, specific trait facets (i.e., alexithymia, empathy, impulsivity, aggression, trait anxiety, and depression), and control variables. Control tests showed that the TSST conditions of the project did not influence the correlations found, which is why they were not included as a predictor in the present research question. Age and pubertal status showed high intercorrelation (*r* = 0.61, *p* < 0.001); thus, only age was considered in further analysis. Group differences in stress parameters between male and female participants were examined using the Mann–Whitney *U* test as a non-parametric test suitable for dichotomous group comparisons. To interpret the pure correlations between stress parameters and personality/temperament/traits, a False Discovery Rate (FDR) correction according to Benjamini and Hochberg (1995) was applied. The significance level was set as α = 0.05.

## 3 Results

Table 2 presents the psychometric characteristics regarding personality, temperament, and specific trait aspects, as well as control variables of the sample.

**Table 2:** Psychometric characteristics.

	***N***	***M* (*SD*)**	**Range sample**	**Range questionnaire**
**Personality (BFI-10)**				
Neuroticism	72	2.75 (1.12)	1.0–5	1–5
Extraversion	72	3.35 (1.02)	1.0–5	1–5
Openness	72	3.56 (1.08)	1.0–5	1–5
Agreeableness	72	3.59 (0.94)	1.5–5	1–5
Conscientiousness	72	3.39 (0.95)	1.5–5	1–5
**Temperament (JTCI 12–18 R)**				
Novelty seeking	73	25.38 (8.01)	7–45.5	0–60
Harm avoidance	73	21.29 (9.99)	2–49.0	0–52
Reward dependence	73	40.23 (10.00)	17–58.24	0–72
Persistence	73	34.64 (9.14)	10–52.0	0–56
**Specific trait aspects**				
Alexithymia (AQC-G)	72	0.60 (0.29)	0.00–1.30	0–2
Empathy (CASES-G)	73	40.88 (9.87)	19–60	0–60
Anxiety and depression trait (STADI)	73	35.42 (8.26)	21–58	20–80
Impulsivity (BIS-15)	73	29.48 (6.32)	16–44	15–60
Aggression (AQ)	71	52.48 (11.62)	34–88	29–116
**Control variables**				
Trauma (CTQ-SF)	71	31.01 (5.77)	25–51	25–125
Stress Vulnerability (SSKJ 3-8R)	73	19.38 (3.84)	10–25	7–28

In all questionnaires, higher scores represent a higher level of the construct. Missing values resulted from missing items in questionnaires. Single missing items were corrected according to questionnaire instructions. CTQ-SF, childhood trauma questionnaire–short version; STADI, state-trait anxiety-depression inventory; SSKJ, questionnaire for assessment of stress and coping in childhood and adolescence, scale range from 7–28; AQC-G, alexithymia questionnaire for children–German version.

### 3.1 Correlative relationships

First, we analyzed the demographic (age, sex, and type of school) and control (trauma and vulnerability to stress) variables for correlations with all stress parameters. We found that cortisol was significantly correlated with age (CORT AUCg × age: *r* = 0.23, *p* = 0.007; CORT AUCi × age: *r* = 0.20, *p* = 0.018; CORT Delta_increase_ × age: *r* = 0.20, *p* = 0.017). Additionally, alpha-amylase was correlated with age (AMYL AUCi × age: *r* = 0.17, *p* = 0.044; AMYL Delta_increase_ × age: *r* = 0.17, *p* = 0.048). Regarding subjective stress responses, we found that age was correlated with anticipatory stress (VAS_Prep_ Delta_increase_ × age: *r* = 0.21, *p* = 0.025), and vulnerability to stress was correlated with stress response after the TSST (VAS_Stress_ Delta_increase_ × stress vulnerability: *r* = 0.27, *p* = 0.002). These correlations were considered in further analyses. All other examined parameters showed no significant correlations with the stress parameters (all *r* < 0.16 and *p* > 0.063).

Next, we examined correlations of stress parameters with personality, temperament, and specific trait facets. Due to their intercollinearity, personality and temperament were considered separately into the models to examine their respective associations with stress reactivity. Supplement Table A presents the correlations among personality, temperament, and personality specific trait facets. These general intercorrelations between constructs were calculated for exploratory purposes, as they offer additional insights beyond the main scope of the current study. For clarity and conciseness, they are reported in the Supplementary material.

In the next step, we examined the associations with the actual stress parameters for further regression analyses. We found significant correlations between the different stress parameters and the personality/temperament/traits dimensions. All correlations are presented in Table 3. Due to the risk of Type I errors in the interpretation of the correlations, an FDR correction was applied to the correlations between stress parameters and personality/temperament/traits, which is highlighted in Table 3.

**Table 3:** Correlations among stress parameters, personality, temperament, and specific trait facets.

		**N**	**E**	**O**	**A**	**C**	**NS**	**HA**	**RD**	**P**	**ALX**	**A&D**	**EMP**	**IMP**	**AGR**
**CORT**	**AUCg**	0.12	**−**0.22 ^*^	0.00	0.03	0.03	**–**0.09	0.20 ^*^	0.04	**–**0.05	0.01	0.14	0.01	**–**0.01	**–**0.06
	**AUCi**	0.06	**−**0.23 ^**^	0.01	**–**0.01	**–**0.04	**–**0.05	**0.18** ^*^	**–**0.02	**–**0.09	0.08	**0.16** ^*^	**–**0.07	0.06	**–**0.01
	**Delta**_**Increase**_	0.09	**−**0.24 ^**^	0.02	**–**0.02	**–**0.03	**–**0.04	**0.18** ^*^	0.00	**–**0.08	0.06	**0.18** ^*^	**–**0.03	0.06	**–**0.01
**AMYL**	**AUCg**	0.11	**–**0.12	**−0.17** ^*^	**–**0.09	**–**0.11	**–**0.02	0.12	**–**0.13	**–**0.03	0.16	0.15	**–**0.14	0.11	**0.23** ^**^
	**AUCi**	0.14	**–**0.12	0.03	**–**0.03	0.02	**–**0.01	0.13	0.00	**–**0.01	0.10	0.13	**–**0.02	0.05	0.04
	**Delta**_**Increase**_	0.14	**–**0.11	0.01	**–**0.03	0.01	**–**0.01	0.13	**–**0.02	**–**0.01	0.09	0.14	**–**0.03	0.07	0.05
**HR**	**Delta**_**Increase**_	0.00	**–**0.08	0.08	0.12	**–**0.04	**–**0.03	0.07	0.00	**–**0.12	0.11	0.09	**–**0.02	0.05	0.02
**HRV**	**Delta**_**Decrease**_	0.02	**–**0.13	0.06	0.03	0.16	**–**0.08	0.06	0.06	0.11	**–**0.02	0.05	0.12	**–**0.05	**–**0.11
**VAS**_**Prep**_	**Delta**_**Increase**_	0.12	**–**0.06	**–**0.07	0.01	**−0.20** ^*^	**0.19** ^*^	0.26 ^**^	0.05	**−**0.26 ^**^	0.10	**0.23** ^**^	0.02	**0.23** ^**^	0.11
**VAS**_**Stress**_	**Delta**_**Increase**_	**0.22** ^*^	**–**0.03	0.02	0.09	**–**0.07	**–**0.03	0.26 ^**^	0.02	**–**0.14	0.09	0.15	0.01	0.06	0.03

^*^p ≤ 0.050, ^**^p ≤ 0.010. Bold values indicate significant correlations, additionally underlined values remain significant after false discovery rate correction. CORT, cortisol; AMYL, alpha-amylase; HR, heart rate; HRV, heart rate variability; VAS, visual analogue scale; AUCg, area under the curve with respect to ground; AUCi, area under the curve with respect to increase; Prep, preparation phase before stress induction; Post, after stress induction; N, neuroticism; E, extraversion; O, openness; A, agreeableness; C, conscientiousness; NS, novelty seeking; HA, harm avoidance; RD, reward dependence; P, persistence; ALX, alexithymia; A&D, anxiety and depression, trait; EMP, empathy; IMP, impulsivity; AGR, aggression.

### 3.2 Linear regressions examining stress reactions across different stress parameters

For linear regressions, we only used predictors that previously showed a significant correlation with the given stress parameter. In the analysis of sex differences in stress parameters, male participants showed significantly higher cortisol levels (CORT AUCg: *U* = 835.00, *p* = 0.014; CORT Delta_increase_: *U* = 821.00, *p* = 0.022) compared to female participants. No significant sex differences were found for the other physiological or subjective stress parameters (all *p* > 0.228). The detailed coefficients and explained variances of the regressions for personality are shown in Table 4, and those for temperament in Table 5. Across all regressions, significant results were only found with the models regarding subjective stress reactions: for personality. Model-level statistics for significant results: VAS_Prep_ Delta_increase_: *F*_(4)_ = 4.01, *p* = 0.006, and VAS_Stress_ Delta_increase_: *F*_(2)_ = 7.47, *p* = 0.001; for temperament, VAS_Prep_ Delta_increase_: *F*_(6)_ = 3.28, *p* = 0.007, and VAS_Stress_ Delta_increase_: *F*_(2)_ = 6.34, *p* = 0.003. Across those four significant models, 12.9–15.9% of the variance could be explained, which corresponds to a medium-to-large effect size (*d* = [0.77; 0.87]), according to Cohen (1988). Significant predictors were only identified in models of the acute stress reaction immediately after the TSST (VAS_Stress_ Delta_increase_). Regarding personality, Neuroticism and Stress Vulnerability were significant predictors of the subjective stress response following the TSST. Specifically, an one-point increase in Neuroticism was associated with a 0.53-point increase in the subjective stress response, while an one-point increase in Stress Vulnerability led to a 0.18-point increase in the subjective stress response. In the temperament model, only Stress Vulnerability was a significant predictor: as in the personality model, a one-point increase in Stress Vulnerability resulted in a 0.18-point increase in the subjective stress response.

**Table 4:** Results of reduced linear regression models for personality aspects predicting adolescents' stress reactions after psychosocial stress induction.

**Dependent variable**	**Predictor**	***B***	**SE**	***B*** **CI**		**β**	***t***	***p***	***R^2^***
				**Lower**	**Upper**				
CORT AUCg	Age	25.78	25.65	−25.39	76.97	0.13	1.01	0.318	0.04
	Sex	110.20	96.81	−82.98	303.37	0.14	1.14	0.259	
	Extraversion	−51.42	44.48	−140.16	37.33	−0.14	−0.14	0.252	
CORT AUCi	Age	16.07	21.80	−27.43	59.56	0.09	0.74	0.464	0.02
	Extraversion	−30.08	41.15	−112.19	52.03	−0.09	−0.73	0.467	
	Anxiety and depression	6.04	5.03	−4.01	16.08	0.15	1.20	0.234	
CORT Delta_increase_	Age	0.21	0.05	−0.84	1.25	0.05	0.04	0.696	0.03
	Sex	2.34	2.02	−1.64	6.40	0.15	1.18	0.242	
	Extraversion	−0.81	0.94	−2.69	1.07	−0.11	−0.86	0.393	
	Anxiety and depression	0.11	0.12	−0.13	0.34	0.12	0.92	0.364	
AMYL AUCg	Openness	−359.31	267.33	−893.20	174.58	−0.16	−1.34	0.184	0.04
	Aggression	36.21	25.22	−14.16	86.57	0.17	1.44	0.156	
VAS_Prep_ Delta_increase_	Age	0.18	0.10	−0.02	0.37	0.20	1.79	0.079	0.15
	Conscientiousness	−0.18	0.23	−0.64	0.28	−0.11	−0.78	0.437	
	Anxiety and depression	0.03	0.02	−0.02	0.08	0.14	1.18	0.243	
	Impulsivity	0.06	0.04	−0.02	0.13	0.22	1.54	0.128	
VAS_Stress_ Delta_increase_	Neuroticism	0.53	0.26	0.01	1.05	0.23	2.02	**0.047**	0.15
	Stress vulnerability	0.18	0.08	0.03	0.33	0.28	2.41	**0.019**	

*R*^2^ indicates corrected *R*^2^. CORT, cortisol; AMYL, alpha-amylase; AUCg, area under the curve respective to ground; AUCi, area under the curve respective to increase; VAS, visual analogue scale regarding the question “How stressed, burdened, or tensed do you feel at the moment?”; VAS_Prep_, preparation phase; VAS_Stress_, after stress induction. Significant values are indicated in bold.

**Table 5:** Results of reduced linear regression models for temperament aspects predicting adolescents' stress reactions after psychosocial stress induction.

**Dependent variable**	**Predictor**	***B***	**SE**	***B*** **CI**		**β**	***t***	***p***	***R^2^***
				**Lower**	**Upper**				
CORT AUCg	Age	31.25	24.47	−17.55	80.06	0.16	1.28	0.206	0.04
	Sex	69.35	98.76	−127.67	266.36	0.09	0.70	0.485	
	Harm avoidance	5.30	4.63	−3.95	14.54	0.14	1.14	0.257	
CORT AUCi	Age	17.85	21.27	−24.58	60.28	0.10	0.84	0.404	0.01
	Harm avoidance	0.74	5.36	−9.94	11.43	0.02	0.14	0.890	
	Anxiety and depression	6.38	6.56	−6.70	19.46	0.16	0.97	0.334	
CORT Delta_increase_	Age	0.30	0.50	−0.71	1.30	0.07	0.59	0.555	0.02
	Sex	2.08	2.01	−1.93	6.09	0.13	1.03	0.305	
	Harm avoidance	0.04	0.13	−0.22	0.29	0.05	0.28	0.778	
	Anxiety and depression	0.11	0.15	−0.19	0.41	0.12	0.73	0.468	
VAS_Prep_ Delta_increase_	Age	0.17	0.10	−0.03	0.37	0.20	1.74	0.087	0.16
	Anxiety and depression	0.01	0.03	−0.05	0.07	0.04	0.28	0.781	
	Impulsivity	< 0.00	0.05	−0.10	0.10	< -0.01	−0.01	0.993	
	Novelty seeking	0.03	0.03	−0.02	0.09	0.16	1.22	0.226	
	Harm avoidance	0.02	0.03	−0.03	0.08	0.15	0.92	0.361	
	Persistence	−0.04	0.03	−0.10	0.02	−0.22	−1.29	0.201	
VAS_Stress_ Delta_increase_	Harm avoidance	0.05	0.03	−0.01	0.11	0.19	1.58	0.120	0.13
	Stress vulnerability	0.18	0.08	0.02	0.33	0.27	2.22	**0.030**	

*R*^2^ indicates to corrected *R*^2^. CORT, cortisol; AUCg, area under the curve respective to ground; AUCi, area under the curve respective to increase; VAS, visual analogue scale regarding the question “How stressed, burdened, or tensed do you feel at the moment?”; VAS_Prep_, preparation phase; VAS_Stress_, after stress induction. Significant values are indicated in bold.

### 3.3 Power considerations

A *post-hoc* sensitivity analysis (two-tailed α = 0.05, target power = 0.80, *n* = 73) was carried out with G^*^Power 3.1 for (i) the zero-order correlations and (ii) the follow-up multiple regressions.

*Correlations*. With *n* = 73 we were able to detect a Kendall's τ of ≈ 0.21, equivalent to a Pearson *r* of ≈ 0.32— a medium effect according to Cohen (1988). Very small associations (τ < 0.20) may therefore have gone undetected.

*Regressions*. Because the six linear models contained different numbers of predictors (2, 3, and 4), we computed the minimum incremental effect (addition of one focal predictor, controlling for the remaining covariates) that could be picked up with 80 % power: according to Cohen's benchmarks for *f*^2^ (small = 0.02, medium = 0.15, large = 0.35), the study was therefore well powered to detect medium or larger incremental effects (*f*^2^ = 0.12–0.15), whereas very small effects (*f*^2^ < 0.02) would likely remain non-significant. These thresholds contextualise any null findings and provide a concrete basis for future sample-size planning.

## 4 Discussion

In this study, we aimed to investigate how reactivity to psychosocial stress among healthy adolescents is related to their personality and temperamental characteristics. In 73 adolescent participants, we assessed temperament dimensions, personality according to the Big Five model, and various trait facets (anxiety and depression, alexithymia, empathy, impulsivity, and aggression). These factors were analyzed in conjunction with the multifactorial stress response (cortisol, alpha-amylase, heart rate, heart rate variability, and subjective stress) following the induction of psychosocial stress using a laboratory stress paradigm (TSST). Based on relevant relationships, we subsequently tested the respective scales of personality and temperament for the amount of variance they explained in the stress response.

General analysis of personality factors, specific trait facets, and temperament dimensions revealed relationships consistent with previous findings. Harm Avoidance was positively correlated with both Neuroticism and trait anxiety/depressiveness, showing a strong association. This consistency reflects the conceptual overlap among the three constructs, all of which encompass aspects of anxiety. Additionally, we found strong positive associations between Persistence and Conscientiousness, and a negative correlation between Persistence and Impulsivity.

One of the most commonly used stress parameters, salivary cortisol, was negatively correlated with the personality aspect of Extraversion in our sample; participants with higher levels of Extraversion showed lower cortisol reactivity, and vice versa. This is in line with previous findings of a meta-analysis (Luo et al., 2023). However, the stress response could not be explained based on the Extraversion level, possibly due to the small effect size resulting from the present sample size. This assumption aligns with findings from studies among adults, as the reported effects fall within a comparable small range (Luo et al., 2023). Additionally, a significant correlation was found between cortisol levels and the temperament scale of Harm Avoidance, suggesting that individuals with higher Harm Avoidance tendencies exhibited higher cortisol levels, but after controlling for age, sex, anxiety and depression, no associations were observed between the stress response and temperament. This contrasts with the findings of Tyrka et al. (2007), who reported associations between cortisol and the temperament traits of Novelty Seeking and Harm Avoidance in young adults.

Despite the assumption that the SAM system is more strongly correlated with personality (Luo et al., 2023), our adolescent sample exhibited almost no correlations between autonomic nervous system parameters (alpha-amylase, HR, and HRV) and personality or temperament. Only Openness showed a weak positive correlation with the total release of alpha-amylase, but this did not significantly explain the stress response. Given that Openness has shown negligible effects in previous studies (Luo et al., 2023), this weak correlation should be cautiously interpreted and the absence of stronger associations could be due to limited statistical power or multiple testing.

The subjective stress response appears to be most strongly associated with temperament or personality, which is also reflected in our present results. During preparation (i.e., when stress arose in anticipation of the unknown task), participants with lower Conscientiousness reported higher subjective stress. Conversely, Neuroticism was significantly correlated with the actual stress response to the TSST, with higher subjective stress responses associated with higher Neuroticism levels. These results align with previous findings of studies in samples of adults (Luo et al., 2023). In our present sample, the Neuroticism score was the only personality trait that explained variance in the subjective stress response to the TSST. Generally, caution must be exercised when interpreting these exploratory results, as there is a risk of a Type I error. In terms of temperament, Harm Avoidance was positively correlated with subjective stress both before and after the TSST. In a study among young adults, Puttonen et al. (2005) also found that perceived stress was positively associated with Harm Avoidance and, in contrast to our findings, also with Novelty Seeking. We found that Novelty Seeking (positive) and Persistence (negative) were correlated with anticipatory stress in preparation for the task. However, these correlations were very small and none of these aspects were sufficiently strong to explain variance in the stress response. After correcting for multiple testing, only Persistence showed a reliable correlation, while the correlation with Novelty Seeking disappeared. Similarly, Ravaja et al. (2006) reported that anticipatory stress was positively correlated with Novelty Seeking, and also with Harm Avoidance (rather than Persistence, as in our study). Once again, the lack of significance in the expected effects could also be attributed to the limited power of our sample. Among our present findings, Stress Vulnerability emerged as the only significant predictor in our regression analysis for both anticipatory and actual subjective stress, with higher Stress Vulnerability levels being associated with higher subjective stress responses. The Stress Vulnerability assessment determines how intensively children and adolescents react with stress to everyday tensions and problems. It is a subjective measure that measures subjective stress, and is therefore linked to the subjective stress reaction in an acute stressful situation. This indicates the importance of this parameter as a control variable when assessing of subjective stress, especially if group comparisons are made. In addition, future studies may benefit from examining potential interaction effects between Stress Vulnerability and personality (i. e., neuroticism) and temperament traits (i.e., Harm Avoidance), as such interactions could reveal more nuanced mechanisms underlying individual differences in stress reactivity. Due to limitations in statistical power, such moderation analyses were not feasible in the current study, but they represent a promising avenue for future research.

The trait facets revealed that trait anxiety and depression were significantly correlated with both cortisol release and anticipatory subjective stress, albeit very weakly. We also observed high intercorrelation with Harm Avoidance and Neuroticism, precluding further interpretation in this regard. Impulsivity only appeared to be correlated with anticipatory subjective stress; however, the effect was very small and thus of negligible relevance. The weak correlation identified between aggressiveness and alpha-amylase release appears to be similarly negligible. None of these traits were significant predictors of the stress response. Alexithymia and empathy appeared to have no or only a minimal correlations with stress reactivity in our sample. However, alexithymia should still be taken into account when measuring subjective stress, particularly in samples with a potentially high prevalence of alexithymia [e.g., clinical populations (Xiao et al., 2024)], as the ability to accurately report internal states may be impaired, potentially compromising the validity of subjective stress ratings.

Many of the significant findings regarding correlations between multifactorial stress reactions and personality/temperament in adulthood could also be found during the dynamic phase of adolescence. This suggests that personality/temperament influences the stress response early in life. Even if personality and temperament factors do not substantially account for variance in the stress response, they should be incorporated as important factors in the transdisciplinary stress model from an early age. Since chronic stress during childhood and adolescence can have negative effects on mental and physical health even into adulthood (Fox et al., 2010), identifying stress-prone personality or temperament profiles could serve as a foundation for prevention, for example, by promoting resilience in these young individuals. In this context, initial findings from studies in young adult populations suggest that interventions such as progressive muscle relaxation can effectively reduce physiological stress markers such as salivary cortisol (e.g., Špiljak et al., 2024). While further research is needed to assess the applicability and efficacy of such interventions in younger adolescents, these results point to promising avenues for early, personality-informed prevention. Integrating psychophysiological screening with targeted support strategies, including stress management programs adapted to individual temperament profiles, could help mitigate maladaptive stress trajectories before they become entrenched.

### 4.1 Limitations

Several limitations of this study must be considered when interpreting the results. Adolescents are still undergoing personality development, which can influence the stability of the measured dimensions (Klimstra et al., 2009). However, this aspect appeared to be negligible in the present study, as it had a cross-sectional design, and stress reactivity was measured using current personality characteristics. The BFI-10 was primarily chosen for its brevity. Due to the concurrent publication of the BFI-K KJ (Kupper et al., 2019), and the early planning phase of our study, we were not yet aware of this more age-appropriate instrument. Future studies should take this more suitable alternative into account. However, the ultra-brief nature of the BFI-10 entails limitations regarding the internal consistency and depth of personality assessment. This trade-off between brevity and psychometric precision should be considered when interpreting the results. Additionally, stress was induced using two different versions of the TSST. Although a significant stress response was evoked with both versions (Ecker et al., 2024), it still represents an irregularity in the methodology, and should be avoided in future research. For our present analysis, we decided to combine the two groups to attain a sufficiently large sample size for investigating personality aspects in adolescents. Nevertheless, our sample size remains fairly small for research in the area of personality, which is why both the significant results and non-findings reported here are primarily intended to serve as preliminary indications in the area of adolescent personality/temperament development. Future studies should aim to increase the sample size, even though this is associated with significant effort, costs, and ethical concerns regarding stress induction in experimental approaches within the field of childhood and adolescence. To better contextualize the present findings, we conducted a *post-hoc* sensitivity analysis. This revealed that with the current sample size (*n* = 73), only medium to large effects could reliably be detected in correlation and regression analyses. Thus, non-significant findings should be interpreted with caution, as smaller but potentially meaningful effects may have gone undetected. Finally, other aspects of the larger research project, particularly a short relaxation intervention following the TSST, may have theoretically influenced physiological responses. However, as previously published (Schleicher et al., 2024), this intervention did not significantly affect acute stress reactivity. To minimize potential confounding, we excluded recovery periods during the preprocessing of stress parameters. This, in combination with the lack of measurable intervention effects, supports our decision to analyze all participants jointly, regardless of group assignment.

### 4.2 Strengths

The present study also has some strengths that must be highlighted. Firstly, the study comprised a well-characterized sample of healthy adolescents, clinical abnormalities were ruled out, and various control variables were collected. Additionally, both the personality and temperament aspects were assessed using valid and reliable measures, enabling an overview of possible associations between the two prevailing models in relation to their explanatory power regarding stress reactivity. Moreover, the stress reaction was recorded using a multimodal approach, allowing for interpretation on a broad range of biobehavioral measures using a uniform methodology.

### 4.3 Conclusion

While the findings of this study may be preliminary, it is one of the first to explore associations between a multifactorial stress response to an acute psychosocial stressor and aspects of both personality and temperament in healthy adolescents aged 11–17 years. The personality trait Extraversion, and the temperament dimension Harm Avoidance, showed correlations with the stress response in a healthy adolescent sample after stress induction. Harm Avoidance was associated with both subjective stress and cortisol levels, while Extraversion showed a negative correlation with the cortisol response. These results coincide with previous findings in samples of adult participants, indicating that, according to the transdisciplinary stress model, dispositional factors of personality or temperament may be consistently linked to stress responses from an early age.

## Data Availability

The datasets generated and/or analyzed during the current study are openly available on the Open Science Framework (OSF; DOI: https://doi.org/10.17605/OSF.IO/6C3QG).
