# Supplementary material for: Temperament and personality: preliminary evidence of possible relationships with multifactorial stress reactivity in healthy adolescents
Source: Front Psychol. 2025 Jul 22;16:1613000. doi: 10.3389/fpsyg.2025.1613000 (PMC12323739; doi:10.3389/fpsyg.2025.1613000)
Supplement: Supplementary file 1 [file Table_1.docx]

# Supplemental Material

**Supplement TABLE A** Correlations among personality, temperament, and specific trait facets.

*Note:* The intensity of grey shading increases with increasing correlation strength. **p* ≤ 0.050, ***p* ≤ 0.010.

*Abbreviations:* N, Neuroticism; E, Extraversion; O, Openness; A, Agreeableness; C, Conscientiousness; NS, Novelty Seeking; HA, Harm Avoidance; RD, Reward Dependence; P, Persistence; ALX, Alexithymia; A&D, Anxiety and Depression, Trait; EMP, Empathy; IMP, Impulsivity; AGR, Aggression.
